# P-1857. Across Five Aprils: A single-center, retrospective pilot study of Outpatient Parenteral Antimicrobial Therapy (OPAT) during the COVID-19 pandemic

**DOI:** 10.1093/ofid/ofaf695.2026

**Published:** 2026-01-11

**Authors:** Hanna Bertucci, Lacy Simons, Muhammad Dhanani, Anne Kurze, Kelly E R Bachta

**Affiliations:** Northwestern University, Chicago, IL; Northwestern University, Chicago, IL; Feinberg School of Medicine, Northwestern University, Chicago, Illinois; Northwestern Memorial Hospital, Chicago, Illinois; Northwestern University, Chicago, IL

## Abstract

**Background:**

Outpatient parenteral antimicrobial therapy (OPAT) refers to the administration of parenteral antimicrobial agents in an outpatient setting and has revolutionized provision of intravenous (IV) therapy to patients with complex infections. By allowing stable patients with deep-seated infections to be discharged early or avoid hospitalization, OPAT is cost-efficient, reduces the risk of nosocomial infection, and maximizes patient comfort. Conversely, OPAT patients are at increased risk of complications due to reduced supervision, and antibiotic overuse is of concern. Despite this, OPAT practices are largely uncharacterized. While 100-150 patients at Northwestern Memorial Hospital’s (NMH) Chicago campus are monitored on OPAT at any time, patient demographics, antibiotic use, and outcomes remain untracked. We aim to assess the OPAT program at NMH before and during the COVID-19 pandemic, examining changes in readmissions, adverse events, administrative burden, and access to care.

**Methods:**

We analyzed outcomes for 229 patients discharged from NMH Chicago on OPAT in the month of April during 2019-2023. Primary data included demographic information, infection type, antibiotic course, and readmission and complication rates, with statistical analyses executed by comparison mechanism.

**Results:**

Each April, 37-53 patients were discharged on OPAT. The cohort was predominantly white (61.1%) and male (58.0%) with a mean age of 61.4 years. Statistically significant variation by race was seen in 2020, with white patients comprising 78.4% of the cohort (p = 0.031, Chi-squared). Overall 90-day readmission was 49%, with 69% of first readmissions unplanned. 90% of readmissions in 2023 were unplanned. 90-day mortality was 6.0% overall, and 13.5% in 2020. Differences in mortality did not reach statistical significance (p=0.19, ANOVA). Four patients were lost to follow-up, and five transferred care to other institutions. Analysis continues for infection type, antibiotic use, mortality, and complications.

**Conclusion:**

There is a need for a highly coordinated multidisciplinary team approach and close monitoring of OPAT patients. With 12,000 patients projected to complete OPAT therapy at NMH by 2030, this project offers key pilot data to improve Northwestern’s OPAT practices system-wide.

**Disclosures:**

All Authors: No reported disclosures

